# Impact of Hot Water Extraction on the Chemical Composition of Hemp (*Cannabis sativa* L.)

**DOI:** 10.3390/ma18194576

**Published:** 2025-10-02

**Authors:** Kamil Roman, Monika Marchwicka

**Affiliations:** 1 Department of Technology and Entrepreneurship in the Wood Industry, Warsaw University of Life Sciences—SGGW, Nowoursynowska 159, 02-787 Warsaw, Poland; kamil_roman@sggw.edu.pl; 2 Department of Wood Science and Wood Protection, Warsaw University of Life Sciences—SGGW, Nowoursynowska 159, 02-787 Warsaw, Poland

**Keywords:** hemp, HWE, lignocellulosic biomass, chemical composition, biorefinery

## Abstract

An investigation of the effect of intense Hot Water Extraction (HWE) on the chemical properties and processability of shredded hemp stalks (*Cannabis sativa* L.) is presented in this study. The chemical composition of untreated hemp was compared to that of hemp subjected to V and XV successive HWE cycles. This study investigated changes in selected chemical compounds, such as extractives, lignin, cellulose, ash, and monosaccharides such as glucose and xylose. Additionally, post-HWE liquids were analyzed. Lignin content was determined by the UV–VIS spectrophotometry method, whereas monosaccharides (glucose, xylose) and inhibitors (formic acid, acetic acid, levulinic acid, ethanol, 5-(hydroxymethyl)furfural, and furfural) were identified by HPLC. Extractives and ash were effectively removed by the HWE process, decreasing from 3.2 to 2.0% and from 3.9% to 2.7%, respectively. The reduction in acid-soluble lignin was an important finding, indicating a selective modification of the lignin matrix. By the end of V cycles, xylose content in the liquid phase significantly increased from 117.9% to 19.4%, indicating a reduction in hemicelluloses. The cellulose content of the solid material rose from 42.9% to 46.2% at the end of XV cycles.

## 1. Introduction

Restrictions on fossil fuel resources and their increased environmental impact make reducing greenhouse gas emissions essential. Despite an increase in energy demand, conventional fossil fuels remain the primary source [1,2]. In response, biomass has been increasingly utilized for heat and power and can also be processed to yield valuable liquid energy carriers [3]. Replacing fossil fuels with energy crops may be an effective way to reduce carbon emissions [4,5]. Identifying energy crops suitable for widespread use is crucial to meet the global energy demand. Beyond lowering greenhouse gas emissions, energy crops also offer the potential for carbon dioxide storage. Climate change can be mitigated by incorporating renewable energy sources with lower greenhouse gas emissions than fossil fuels, which contribute to greenhouse gas emissions [6]. Among these sources, agricultural solid biomass, a plant product, represents significant potential. This category primarily includes agro-industrial waste, energy crops explicitly cultivated for energy, and crop leftovers [7,8,9].

Among the promising plants in this context is hemp [10,11], which is recognized for its high cellulose content and fast growth. This makes it a compelling candidate for biofuel and biorefinery applications [12,13], especially as a feedstock for second-generation bioethanol. Plants like hemp are among the oldest plant-based sources of natural materials, valued for their high tensile strength and environmental benefits [14]. This unique property encourages companies to explore new applications for plant materials. Industrial hemp products are durable, sustainable, and versatile, making them attractive raw materials for textile, construction, and automotive use. Lignocellulosic biomass, predominantly composed of cellulose, hemicelluloses, and lignin, is increasingly considered a suitable feedstock for biofuel production [15,16,17]. The content of cellulose, hemicellulose, and lignin is essential when considering the energy potential of lignocellulosic biomass. The use of hemp biomass can be maximized by applying pretreatment processes. Hot water extraction (HWE) is a method capable of modifying the chemical profile of biomass for use in biorefineries, energy plants, and other applications [18,19,20]. The HWE process affects the content of extractives, lignin, and cellulose [19]. Sustainable hemp biomass utilization requires understanding how successive HWE cycles influence hemp’s chemical structure.

Sustainable hemp biomass utilization requires a thorough understanding of the changes in its chemical structure caused by successive HWE cycles. This study used shredded hemp stalks from a private, certified plantation. The high lignocellulosic content of hemp and its versatility make it a promising candidate for biofuels and biorefineries. Many biofuel producers turn lignocellulosic biomass, mostly cellulose and hemicellulose, into biofuel. Biomass samples were compared with untreated material after V and XV consecutive HWE cycles [19]. As the HWE method affects the components within lignocellulosic materials, this comparison was made to examine changes in extractives, lignin, cellulose, ash, glucose, and xylose [21]. Despite the application of HWE to lignocellulosic biomasses such as wheat straw, corn stover, and miscanthus, little is known about the chemical composition of industrial hemp during repeated extraction cycles. The lignin structure and ash content of hemp differ from those of other crops, which makes this knowledge gap especially important [19,22]. The feasibility of HWE tailored to hemp straw can be evaluated by addressing this issue.

This study investigated the chemical properties and processability of shredded hemp stalks subjected to bioenergy production [23,24]. Although HWE has been reported to alter the chemical composition of hemp biomass in previous research [19], a comprehensive understanding of how successive cycles at low temperatures affect its chemical profile is still lacking. This work aims to assess the suitability of HWE as a sustainable pretreatment for biorefinery applications by investigating how V and XV consecutive HWE cycles influence extractives, lignin, cellulose, and key monosaccharides. Understanding these changes will support the development of more efficient and sustainable processes for converting lignocellulosic biomass into energy and value-added materials [25]. In this study, we provide in-depth chemical and physical analyses of hemp straw after successive HWE cycles, filling a significant knowledge gap and offering a novel approach compared to previous studies focusing on single cycles or other biomasses. We hypothesize that multi-cycle hot water extraction of hemp reduces extractives and ash while preserving cellulose content, enhancing its suitability as a feedstock for biorefineries.

## 2. Materials and Methods

### 2.1. Material Characteristics and Moisture Content

The hemp stalks (*Cannabis sativa* L.), most likely of the industrial hemp cultivar “Białobrzeskie”, which is commonly grown in the Mazovia region, exhibit hygroscopic properties, naturally absorbing and retaining moisture from the surrounding environment due to their inherent chemical composition, just like other lignocellulosic materials. The moisture content of hemp stalks is an important parameter that directly affects the efficiency of the HWE method and the extraction characteristics. Controlling moisture precisely is essential to ensuring the consistency and reproducibility of experiments and optimizing materials for various applications. The biomass was comminuted and meticulously prepared to prepare hemp biomass for HWE treatment. Industrial hemp products are durable, sustainable, and versatile, making them attractive raw materials for textile, construction, and automotive use. The study utilized mechanically comminuted whole industrial hemp stalks grown in Poland, specifically in the Mazovia voivodeship, and harvested at one year of age. The seed heads were removed before processing, and the biomass used was shredded stalks without separation into bast fibers and hurds. It was not necessary to apply retting. It was not possible to confirm the exact cultivar with the supplier, and detailed data on bast/hurd ratios after harvest was unavailable.

In this study, an undried hemp material was used. Drying can lead to the collapse of the biomass porous structure, particularly in lignocellulosic materials such as hemp. This structural collapse reduces the accessibility of the internal cell wall area and slows down or even decreases water penetration during HWE. By maintaining the native, undried structure, the internal pores remain open, increasing water penetration and enhancing extraction efficiency. From an industrial perspective, drying biomass on a large scale is energy-intensive and time-consuming, significantly improving operational costs. Avoiding this step enhances the economic feasibility of the process.

Furthermore, since HWE is water-based, removing any incompatible solvents before treatment is unnecessary. The moisture content of the biomass was determined using the gravimetric method, commonly known as the oven-drying method. To ensure all moisture evaporated, samples were dried at a constant temperature of 104 °C until they reached a stable weight. Each measurement was performed in triplicate to minimize experimental error and ensure the reliability of the results. In this study, the primary aim was to determine if multi-stage HWE can modify the chemical profile of hemp biomass.

### 2.2. The HWE Treatment

The study utilized mechanically comminuted whole industrial hemp stalks grown in Poland, specifically in the Mazovia voivodeship, and harvested at one year of age. Seasonal temperatures generally ranged from 15–24 °C, providing a favorable plant growth climate. The material, mechanically minced and sieved, was prepared for modification and subsequent analysis, including HPLC. The shredded hemp stalks were subjected to the HWE treatment. The pre-shredded material was transferred into a stainless-steel tube containing 10 g of raw material. An extraction was carried out in a 400 mL stainless-steel reactor. The liquid-to-solid ratio in each cycle was 40:1 (*w*/*w*), corresponding to 400 mL of distilled water. The material was not dried before HWE, so internal cellular structures did not collapse, preventing efficient extraction. The particle size (fraction) used for the pretreatment was 0.4–1.0 mm. Distilled water was heated and pressed at a higher pressure to extract soluble substances. A container with distilled water was placed below the reactor to supply purified water. The heated water boiled through the material into the container above. During drying, shredded hemp materials were immersed directly in hot water, without direct steam flow. Steam is generated from boiling water, not liquid water, which this study calls hot water extraction (HWE). Pretreatment was performed at 100 °C for 15 min with a residence time of 10 min.

Based on the objective of this study to sustainably modify hemp biomass for biorefinery applications, the following specific HWE conditions were selected. The selection of V and XV HWE cycles was based on the approach used by Leszczyński and Roman [19], who demonstrated that 1, 2, and 3 cycles were not fully effective in extracting lignocellulosic materials. Following their methodology allows for direct comparison and consistency with research in the field. Building on their findings, we extended the cycles to 5 and 15 for a more intensive extraction treatment. As part of the process, hot water immersion at 100 °C was applied under mild hydrothermal conditions, which was chosen intentionally to preserve the structural integrity of cellulose, a key component in bioethanol production, as well as minimize the formation of inhibitory compounds, such as furfural and HMF, typically found at higher temperatures [26]. It was decided to apply a short residence time of 10 min to maximize efficiency and to concentrate on hemicellulose and extractive removal at the beginning of the process. With both V and XV cycles, we were able to evaluate the impact of both the initial (V cycles) and extended (XV cycles) stages of intense treatment. The multi-stage process allowed us to assess how the repeated removal of labile components affected the biomass overall chemical composition and susceptibility to further processing.

After reaching the set temperature and completing the HWE process, the tube was placed in an ice bath at approximately 0 °C. The liquid fraction after HWE was collected in a volumetric flask. The study compared untreated native material hemp to hemp after V and XV successive HWE cycles to investigate the effects of repeated treatments. Each HWE pretreatment was performed twice to ensure the reliability and reproducibility of the results. Following the HWE process, the material was separated into solid and liquid fractions. Both fractions were preserved by adding 1% sodium azide (NaN_3_) to inhibit microbial growth. Both native hemp material and hemp extracted using hot water were analyzed similarly.

### 2.3. Chemical Composition of Hemp Stalks

#### 2.3.1. Extractive Content

The native hemp material was ground to a 0.4–1.0 mm fraction for all chemical determinations. Hemp after HWE treatment was taken for analysis without additional grinding. The native hemp and the hemp after V and XV cycles of HWE treatment were subjected to extraction. Approximately 3 g of hemp was extracted for 10 h using a Soxhlet extractor with a chloroform–ethanol solvent mixture (93:7 *w*/*w*) [27]. Each determination was made in 3 repetitions. The extracts were subsequently concentrated under reduced pressure and dried to constant weight. The yield of extractives was calculated based on the initial dry mass of the hemp samples.

#### 2.3.2. Cellulose Content

The cellulose content was determined using extractive-free material. The cellulose content was analyzed in the native hemp and hemp after V and XV cycles of HWE treatment. The cellulose content was determined by the Kürschner–Hoffer method [28,29]. This method treated biomass with a nitric acid and ethanol mixture for 3 cycles to remove lignin and hemicellulose. The remaining residue, mainly cellulose, was then collected, dried, and weighed to quantify the cellulose content. Each determination was conducted 3 times. Results are expressed as a percentage of cellulose in relation to the dry weight of the unextracted sample.

#### 2.3.3. Lignin Content

The amount of lignin in native hemp and hemp after V and XV HWE cycles was measured to examine the effects of repeated extractions on the content and composition of lignin. The lignin content was determined using extractive-free material. This determination separated the lignin into two primary fractions: acid-insoluble lignin (AIL) and acid-soluble lignin (ASL). The AIL content was determined by the Tappi method utilizing 72% sulfuric acid—TAPPI 222 om-02 [30]. The AIL demonstrates the part of lignin that is preserved and resistant to acid hydrolysis. It was quantified by applying acid hydrolysis to the material, from which the solid residues, reflecting the lignin that did not dissolve, were gathered and weighed. The ASL content was determined according to TAPPI UM 250 [31]. The ASL indicates the lignin that disintegrates and dissolves during the acid treatment, providing information on the degradability and removal of lignin throughout the HWE procedure. It was assessed by examining the liquid produced after acid hydrolysis, where the dissolved lignin was measured using UV–VIS spectrophotometry at a wavelength of 205 nm. Three repetitions were made to determine the AIL and ASL content. The total lignin content was calculated as the sum of ASL and AIL.

#### 2.3.4. Glucose and Xylose Content

Following the procedures by Saeman et al. (1945) [32] and Kačík and Solár (1999) [33], the glucose and xylose content in the native and pre-treated hemp samples was determined using an acid hydrolysis procedure using 72% sulfuric acid (H_2_SO_4_). The extractive-free material was exposed to 72% H_2_SO_4_ at 30 °C for 1 h (a step intended to disintegrate the complex lignocellulosic matrix, causing the tightly bound cellulose and hemicellulose structures to become more available for further hydrolysis). The initial treatment was followed by heating to its boiling point for 4 h in water-diluted 3% sulfuric acid. After this time, the solid residues were carefully separated from the liquid phase, which included the hydrolyzed sugars. The concentrations of monosaccharides, such as glucose and xylose, were determined in the liquid phase.

Before HPLC analysis, the samples were neutralized to a pH of approximately 5. For ten minutes, the samples were centrifuged at 12,000 rpm. After centrifugation, any remaining dust contaminants were eliminated by filtering each sample through a nylon syringe filter with a 0.2 μm porosity. A Shimadzu (Shimadzu Corp., Kyoto, Japan) liquid chromatograph, which included a CBM-20A control module, RID-10A refractometer detector, CTO-20A oven, DGU-20A degasser, and LC-20AD pump, was used to analyze the glucose and xylose in the hydrolysate. Software LC Solution v.1.21 SP1 was used to analyze chromatograms. The following conditions were applied to acid-hydrolyzed samples:

- eluent: redistilled water
- column: Rezex™ RHM-Monosaccharide H+ Phenomenex,
- oven temperature: 80 °C,
- flow rate: 0.6 mL·min^−1^,
- injection volume: 20 µL.

The glucose and xylose calibration solutions were prepared according to the concentrations of 4.16, 2.08, 1.39, 1.04, 0.46, and 0.38 mg·cm^−3^. Calculated calibration curve equations for glucose and xylose:

y = 3.642 × 10^−10^ × x; R^2^ = 0.9895 (xylose)

y = 4.421 × 10^−10^ × x; R^2^ = 0.9697 (glucose)

#### 2.3.5. Ash Content

The ash content of hemp biomass was determined using a laboratory electric muffle furnace SNOL 8.2/11. A sample of approximately 2 g of dried hemp was weighed. The samples underwent an initial thermal treatment, which involved heating them to 100 °C and keeping them at this temperature for 1 h. In the next step, the temperature increased to 200 °C and was kept for 1 h. This process was repeated at 300 °C, 400 °C, and 500 °C, and each heating step was maintained for 1 h. With each temperature increase, the remaining organic material was able to burn gradually. Once the temperature reached 500 °C, it was increased to 600 °C and maintained for 6 h. This prolonged exposure to elevated temperatures was crucial for the complete combustion of the material, allowing for an accurate measurement of ashes. The samples remained at 100 °C for 4 h, then were placed in a desiccator and allowed to cool before weighing. The ash content was determined based on the residual ash and a comparison with the samples’ original dry weight.

### 2.4. Post-HWE Liquid Analysis

#### 2.4.1. Glucose, Xylose, and Other Degradation Products in Post-HWE Liquid

Following the treatment, the liquid fraction was collected for chemical characterization. This liquid fraction was analyzed using HPLC to detect key sugars such as glucose and xylose, which indicate the solubilization of mono-, oligo-, and polysaccharides. In addition to sugars, the analysis included organic acids (such as acetic acid, formic acid, or levulinic acid) that may be released during the hydrolysis of biomass components. Furthermore, the breakdown of pentoses and hexoses from hemp biomass under hydrothermal conditions can lead to the formation of byproducts such as furfural and 5-(hydroxymethyl)furfural (HMF). A chemical analysis of the liquid fraction after HWE of hemp biomass was conducted to determine its composition. Quantitative determination of monosaccharides (glucose, xylose), other degradation products (5-(hydroxymethyl)furfural (HMF), furfural), and organic acids (acetic, formic, levulinic, and ethanol) was performed using Shimadzu HPLC. Samples were centrifuged at 12,000 rpm, filtered, and analyzed using a refractive index detector (RID), with a Rezex RHM column.

The following conditions were applied to post-HWE liquid samples:

- eluent: 0.005 M H_2_SO_4_
- column: Rezex™ RHM-Monosaccharide H+ Phenomenex,
- oven temperature: 80 °C,
- flow rate: 0.6 mL·min^−1^,
- injection volume: 20 µL.

A series of standards containing the analyzed substances was prepared. This step was crucial in ensuring the accuracy of our results.

#### 2.4.2. Lignin in Post-HWE Liquid Analysis

UV–VIS spectrophotometry was used to determine the lignin content in the post-HWE liquid. This method may be affected by other phenolic compounds, resulting in overestimation of lignin levels. However, the method was selected because it is fast, reproducible, and widely accepted for screening aromatic compounds released during hydrothermal treatments [34]. It provides valuable insight into the process by comparing the effectiveness of different HWE cycles. The soluble lignin content was determined by UV–VIS spectrophotometry at a wavelength of 205 nm. Samples were diluted appropriately to minimize interferences from degradation compounds and fit within the calibration curve’s linear range. Lignin concentration was calculated using a standard calibration curve and an appropriate extinction coefficient at 205 nm—110 L·g^−1^·cm^−1^ [31]. The soluble lignin content in the liquid phase was quantified using a UV–VIS spectrophotometric method, and the results are expressed as a percentage relative to the dry mass of the native (untreated) hemp.

### 2.5. Statistical Properties

The significance of the differences observed in the data was assessed using Analysis of Variance (ANOVA) [35]. The ANOVA is a robust statistical tool that determines if there are statistically significant differences between the means of studied groups. Following the ANOVA, a post hoc Duncan’s test was employed to identify which groups were statistically distinct. This approach provided a more nuanced understanding of the results beyond knowing that differences exist. In the context of this study, Duncan’s test is a statistical method utilized for comparing multiple groups after an ANOVA, which helps to mitigate the likelihood of incorrectly concluding differences between groups when no such differences exist [36].

In this study, tables and figures were used in a complementary manner. Tables present exact mean values with standard deviations and indicate homogeneous groups identified by Duncan’s post hoc test, ensuring statistical precision. Figures, in turn, depict the same results visually, facilitating the interpretation of overall trends and illustrating the correlations revealed by the ANOVA analysis. This dual presentation combines statistical rigor with intuitive readability and avoids ambiguity in data interpretation.

## 3. Results

### 3.1. Material Characteristics and Moisture Content

The research investigated hemp as a possible raw material for bioethanol production. The moisture content of the hemp stalks was found to be 7.2% (with a standard deviation of 0.2%). Industrial hemp can greatly benefit bioethanol production and other applications.

### 3.2. Chemical Composition of Hemp Stalks

#### 3.2.1. Extractive Content

The analyses provided crucial data on how HWE modifies the chemical profile of hemp shives, helping to evaluate the material potential for bioethanol applications. The effect of the HWE cycles on hemp extractive content was assessed using a chloroform and ethanol solvent mixture in a Soxhlet apparatus. The extractive content in the native hemp and hemp treated with V and XV cycles of HWE is shown in Table 1.

The extractive content in hemp decreases after the HWE treatment. After V cycles, there is a noticeable decrease in the extractive content. In the first V cycles, extractives rapidly decreased, confirming the effectiveness of HWE in removing soluble, non-structural components. HWE can be an integral step in a biorefinery because these compounds inhibit downstream processes, such as enzymatic hydrolysis and fermentation. Extractive content values for hemp treated with HWE for V and XV cycles show no statistically significant differences. The values remain relatively stable between these two treatment cycles, indicating a plateau in extractive removal. Consistency among replicates was suggested by all measurements having standard deviations within ±0.3%. Between V and XV cycles, this stabilization most likely reflects the readily water-soluble, low-molecular-weight fraction depletion and diffuse mass transfer from the particle interior. The remaining extractives are less accessible or soluble under the applied conditions as extraction proceeds.

**Figure 1:**
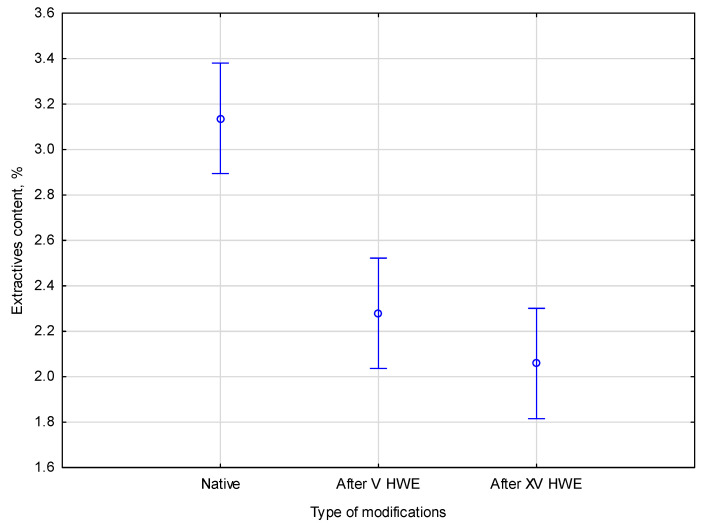
The comparison of extractive content in the native and HWE-treated material. Error bars represent the standard deviation (*SD*) of three replicate measurements.

The study assessed the differences in extractive content between native and HWE-treated material caused by modifications. Statistical analysis of the collected data revealed significant differences in the parameters measured between native and pre-treated hemp biomass. According to the empirical statistics, *F*(2, 9) = 28.191, *p* = 0.00013. The relationship between extractive content in the native and HWE-treated material is presented in Figure 1.

Based on the extractive content comparison shown in the Figure, it is found that the extractive content progressively decreases after the first V HWE cycles, dropping from 3.2 ± 0.2% in native hemp to 2.2 ± 0.3%, before stabilizing at 2.0 ± 0.1% after XV cycles. Therefore, most easily soluble extractives are removed in the early stages of treatment. According to Duncan’s post hoc test, the native material and the HWE-treated samples showed a significant difference (*p* < 0.05) between the two homogeneous groups. This confirms that the figure values do not result from substantial differences between the V cycle and XV cycle groups.

#### 3.2.2. Cellulose Content

To assess the impact of multi-stage HWE on the main polysaccharide component, cellulose, detailed analyses were conducted. The percentage of cellulose in native hemp and hemp treated with V and XV cycles of HWE is presented in Table 2.

The cellulose content in hemp increases with the number of HWE cycles applied. After V cycles, the extractive content already increased noticeably. The change is approximately 4.2% for the cellulose content of native hemp. With each additional HWE cycle, hemp’s cellulose content increases. Extractive content values for hemp treated with HWE for V and XV cycles also show statistically significant differences. The change is approximately 7.7% for the cellulose content of native hemp and 3.4% for the cellulose content of hemp after V HWE cycles. Consistency among replicates was suggested by all measurements having standard deviations within ±0.8%.

**Figure 2:**
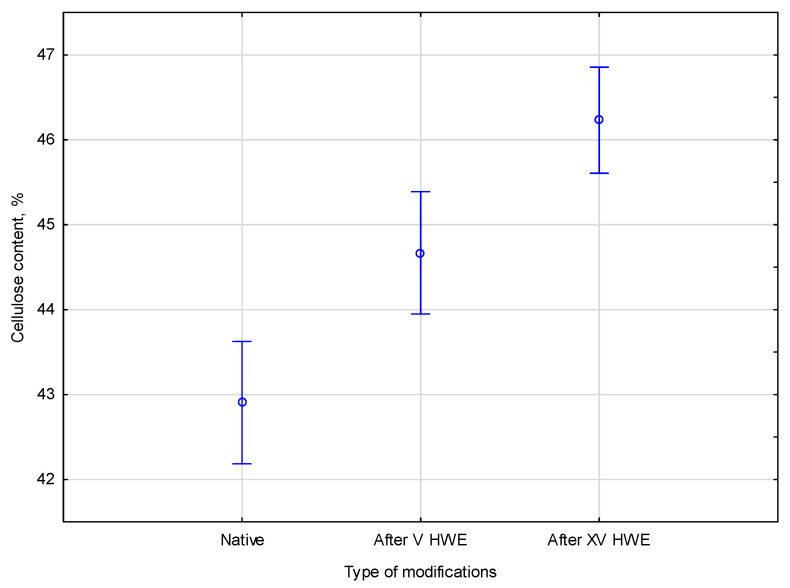
The comparison of cellulose content in the native and HWE-treated material. Error bars represent the standard deviation (*SD*) of three replicate measurements.

The samples were studied in their raw form and after being treated with HWE. To understand the effects of these treatments, the cellulose content was compared after V and XV cycles of HWE. The study assessed differences in cellulose content between the native and HWE processes, with a *p*-value of 0.00025. Statistical analysis of the collected data showed significant differences in the parameters measured. The empirical statistics were equal (*F*(2, 7) = 34.050). The cellulose content in the native and HWE-treated material is presented in Figure 2.

An additional post hoc analysis was performed to confirm the trends shown in the figures. In native hemp, the cellulose content increased from 42.9% to 44.7% after V HWE cycles and to 46.2% after XV cycles. During treatment, hemicelluloses and extractives are removed, leading to the gradual enrichment of cellulose. Each of the parameters belonged to one of the three different homogeneous groups. As determined by the alpha significance level, *p* was below the significance threshold 0.05. Accordingly, the native, V cycle, and XV cycle groups all show statistically significant differences in cellulose content, and Duncan’s test confirms their homogeneity.

#### 3.2.3. Lignin Content

The effect of the number of HWE cycles on acid-insoluble lignin (AIL) and acid-soluble lignin (ASL) content was compared. The AIL content was determined by removing polysaccharides by sulfuric acid hydrolysis. The ASL content was determined in post-hydrolysis liquid with UV–VIS spectrophotometry. The content of lignin in native hemp and hemp treated with V and XV cycles of HWE is presented in Table 3.

HWE pre-treatment causes a decrease in the ASL content in hemp. There is already an apparent decrease in the ASL content after V cycles. There are no diametric variations between the ASL content values of hemp treated with HWE for V and XV cycles. Between these two treatment cycles, the results stay comparatively constant. The homogeneous group with HWE cycles statistically differed from the native-material homogeneous group. All measurements had standard deviations within ±0.4%, indicating consistency between multiple measurements.

Due to material loss during the HWE treatment, hemp’s AIL content slightly increases because of the HWE pre-treatment. Following the V and XV cycles, the AIL content is like that of the natural hemp material. The ASL content values of hemp treated with HWE for V cycles do not differ in a statistically significant way. Similarly, this is the case between V and XV HWE cycles. The above statistical situation created two homogeneous groups in this test. The outcomes remain relatively consistent between the two treatment cycles. Standard deviations for every measurement were within ±0.6%, suggesting that measurements were consistent.

Even though total lignin content remained statistically constant during HWE, the composition of its fractions shifted significantly. After V cycles, the acid-soluble lignin (ASL) content decreased, while the acid-insoluble lignin (AIL) content showed no statistically significant change. The percentage content refers to the weight of the material after pretreatments, where the material loss has occurred. Statistical analysis was conducted to determine how the number of HWE cycles affects the content of acid-insoluble lignin (AIL) and acid-soluble lignin (ASL). To analyze the impact of these treatments, the lignin content was compared after V and XV cycles of HWE.

**Figure 3:**
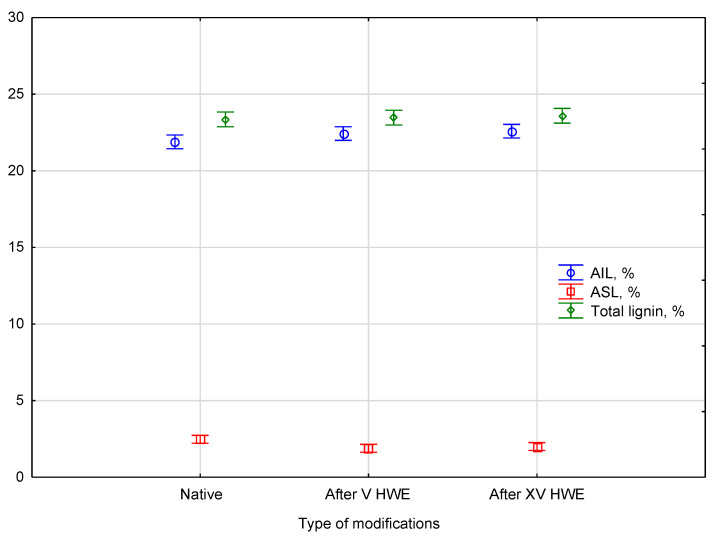
The comparison of lignin percent content between the native and HWE-treated material. Error bars represent the standard deviation (*SD*) of three replicate measurements.

ASL decrease suggests that loosely bound lignin fragments have been solubilized and removed. At the same time, relative increases in AIL indicate a concentration of the more resistant structural lignin in the solid residue. By selectively modifying the lignin matrix, HWE can enhance the accessibility of polysaccharides for enzymatic degradation. Based on a *p*-value of 0.10081, no significant differences were found between types measured in percent content. The empirical statistic, *F*(6, 14) = 2.2357. The comparison of lignin percent content between the native and HWE-treated material is presented in Figure 3.

Statistical analysis using a post hoc test, such as Duncan’s test, confirmed that there were no statistically significant differences in total lignin content between native and HWE-treated samples. The total lignin parameters consistently belonged to a single homogeneous group, indicating the content remained stable throughout the process. In numerical terms, total lignin content was 23.4 ± 0.4% in native hemp, 23.5 ± 0.5% after V cycles, and 23.6 ± 0.3% after XV cycles, confirming the statistical stability of this parameter. The analysis also revealed a shift in the composition of lignin fractions within this group: the content of acid-soluble lignin (ASL) decreased from 2.6 ± 0.4% in native hemp to 1.9 ± 0.2% after V cycles and 2.0 ± 0.1% after XV cycles, while the content of acid-insoluble lignin (AIL) increased slightly from 21.9 ± 0.2% in the native material to 22.4 ± 0.6% and 22.6 ± 0.3% after V and XV cycles, respectively. These changes were observed as early as the V cycle of HWE. The redistribution between ASL and AIL fractions, while not affecting the overall lignin content, is indicative of the solubilization of less stable lignin fragments during hot water extraction.

#### 3.2.4. Glucose and Xylose Content

Hemp biomass underwent HWE pre-treatment to enhance the accessibility of structural carbohydrates. Following the HWE process, the remaining solid fraction was subjected to acid hydrolysis using sulfuric acid to break down polysaccharides (cellulose and hemicelluloses) into their monomeric sugars. Subsequently, the hydrolysate was analyzed using HPLC to quantify the glucose and xylose concentrations, representing cellulose and hemicellulose content. For comparative purposes, untreated (native) hemp was also subjected to the same acid hydrolysis, allowing evaluation of the effect of HWE pre-treatment on sugar yield and biomass composition. The content of glucose and xylose in native hemp and hemp treated with V and XV cycles of HWE is presented in Table 4.

The glucose content in the samples, expressed as a percentage of dry biomass, showed consistent values across replicates. The measured glucose contents were 47.6%, 47.9%, and 46.9%, corresponding to native hemp and samples subjected to V and XV cycles of HWE, respectively. These results show relatively small differences in glucose content between untreated and HWE-treated hemp biomass, suggesting that the HWE treatment had a minimal impact on the cellulose. The glucose content remained statistically unchanged across all samples, indicating that the HWE process effectively preserved the cellulose structure. The low standard deviation in the native material (0.4%) reflects high sample homogeneity, whereas the increased variability observed after V (1.1%) and XV (1.2%) HWE cycles may point to a gradual reduction in sample uniformity because of the extraction process.

The low standard deviation observed in the native (untreated) material was only 0.4% and it indicates a high degree of homogeneity within the sample. The composition was very consistent across different portions of the sample before any treatment was applied. However, after 5 and 15 cycles of HWE, the standard deviation increases to 1.1% and 1.2%, respectively. This increase suggests that the extraction process is gradually introducing more variability into the sample. The uniformity that was initially present was reduced, probably because different components were removed from the biomass at slightly different rates or in uneven ways across the sample. This can result in regions that are chemically or structurally altered to different extents, depending on how each part of the material responds to repeated extraction cycles. However, given that the cellulose content increases while the glucose content remains stable, it can be concluded that some glucose originally present in hemicelluloses has been removed. This suggests a decrease in the overall hemicellulose content.

Pretreatment aims at removing hemicelluloses, opening the biomass structure, and increasing accessibility of cellulose during enzymatic hydrolysis. The xylose content in the samples, expressed as a percentage of dry biomass, also showed relatively consistent values across replicates. The measured xylose contents were 17.9%, 19.4%, and 18.8%, corresponding to native hemp and samples subjected to V and XV cycles of HWE, respectively. These results suggest a slight increase in xylose content following V and XV cycles of the HWE process. The low standard deviations across all samples, particularly after V (0.2%) and XV (0.3%) cycles of HWE, indicate that the treatment did not significantly affect the overall uniformity of the xylose distribution in the biomass. The xylose content of the dry biomass increased from 17.9% to 19.4% after V cycles due to the removal of other compounds, resulting in a higher concentration of the remaining components. A reduction in xylose was observed during the HWE process with XV cycles compared to treatment with V cycles, indicating hemicellulose breakdown.

**Figure 4:**
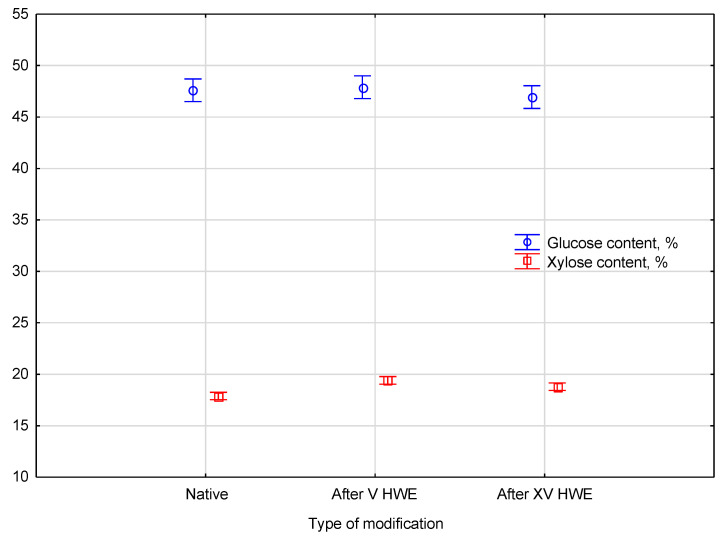
The comparison of the l-glucose and xylose content between the native and HWE-treated material. Error bars represent the standard deviation (*SD*) of three replicate measurements.

Statistical analysis revealed that the HWE treatment had a varied impact on the glucose and xylose content of the material. The glucose content across all samples—native, after V cycles, and after XV cycles of HWE—was found to be consistent, as it was classified into a single homogeneous group. This finding indicates that the HWE process did not introduce any statistically significant differences in glucose content. In contrast, a prominent effect of the treatment was observed for xylose, where the analysis divided the samples into three distinct homogeneous groups. This result confirms that the xylose content changed in a statistically significant manner depending on the number of HWE cycles, highlighting its sensitivity to the extraction process. A detailed comparison of the glucose and xylose content between the native and HWE-treated material is presented in Figure 4.

Based on the ANOVA analysis, the influence of HWE treatment on the monosaccharide content was evaluated. Results indicated that glucose content remained stable in all groups, with values of 47.6 ± 0.4% in native hemp, 47.9 ± 1.1% after V cycles, and 46.9 ± 1.2% after XV cycles, indicating that cellulose structure remained intact even after multiple extraction cycles. All these samples were classified into a single homogeneous group. By contrast, xylose content showed statistically significant differences depending on the number of extraction cycles. The content increased from 17.9 ± 0.4% in native material to 19.4 ± 0.2% after V cycles, followed by a slight decrease to 18.8 ± 0.3% after XV cycles. As a result of the post hoc test, three distinct homogeneous groups were identified, confirming the strong effect of HWE on hemicelluloses. The Wilks’ Lambda statistic of 0.12879, with an *F*(4, 16) value of 7.1458 and a *p*-value of 0.00169, confirmed the statistical significance of these differences. The observed changes indicate that while cellulose remained undisturbed during HWE, the hemicellulosic fraction was progressively removed and redistributed, resulting in measurable modifications of the chemical profile and processing efficiency of hemp biomass. These findings highlight the selective nature of hot water extraction, which enhances cellulose enrichment while altering hemicellulose distribution, an effect of importance for future biorefinery applications.

#### 3.2.5. Ash Content

The solid fraction obtained after the HWE process was analyzed for ash content, representing the total mineral content remaining in the biomass after combustion. For comparison, native (untreated) hemp was also included in the analysis. The objective of this procedure was to assess the effect of HWE on the inorganic fraction of the biomass, which can influence further processing, such as combustion, or inhibit enzymatic hydrolysis efficiency in biochemical conversion processes. The percentage of ash in native hemp and hemp treated with V and XV cycles of HWE is presented in Table 5.

The ash content in the samples, expressed as a percentage of dry biomass, showed a decreasing trend after V and XV cycles of HWE. After V cycles, the ash content has already decreased noticeably. The measured ash contents were 3.9%, 3.1%, and 2.7%, corresponding to native hemp and samples subjected to V and XV cycles of HWE, respectively. These results indicate a progressive reduction in mineral content with increasing cycles of HWE, likely due to the leaching of soluble inorganic compounds during the treatment.

**Figure 5:**
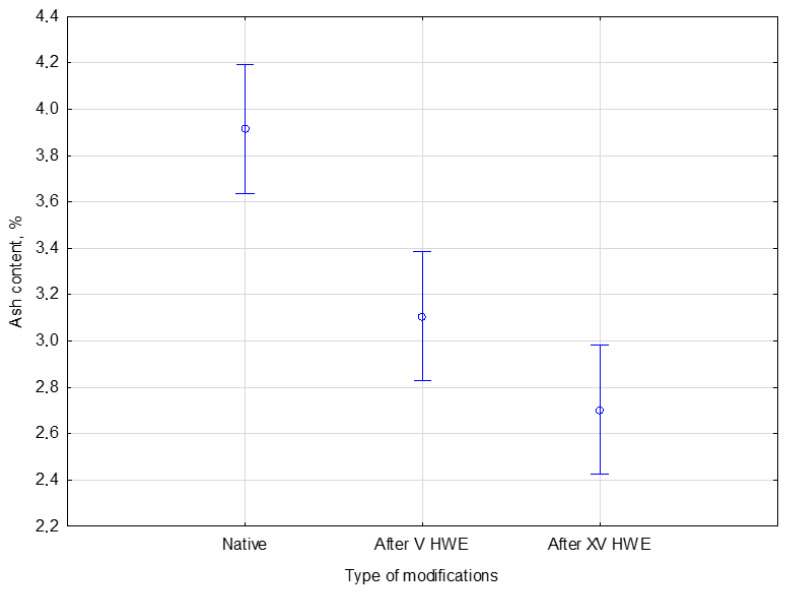
The comparison of ash content in the native and HWE-treated material. Error bars represent the standard deviation (*SD*) of three replicate measurements.

Analysis of the ash content in the solid fraction of biomass, including native hemp, was conducted to determine the total mineral content remaining after combustion. This content is a crucial parameter for assessing the material’s purity and suitability for various applications. To precisely evaluate the impact of HWE treatment on the inorganic fraction of the biomass, a statistical analysis was performed. This study specifically compared the ash content in native hemp with that of samples subjected to both V and XV HWE cycles, with the objective of understanding how the treatment duration influences the mineral composition. The ash content in the native and HWE-treated material is presented in Figure 5.

The Duncan post hoc test indicated that HWE treatment significantly affected the mineral (ash) content of hemp biomass. Native hemp contained 3.9 ± 0.2% ash, which decreased to 3.1 ± 0.1% after V cycles and further to 2.7 ± 0.3% after XV cycles. According to the test statistics, this effect was significant with *F*(2, 6) = 29.288 and *p* = 0.00080. The post hoc analysis confirmed that each treatment level—native, V, and XV cycles—belonged to a different homogeneous group. The results demonstrate that the decline in ash was not random but statistically significant. The above figure shows a clear downward trend in the mineral fraction of hemp biomass with each HWE cycle, indicating increased leaching of soluble inorganic compounds.

### 3.3. Post-HWE Liquid Analysis

#### 3.3.1. Glucose, Xylose, and Other Degradation Products in Post-HWE Liquid

**Figure 6:**
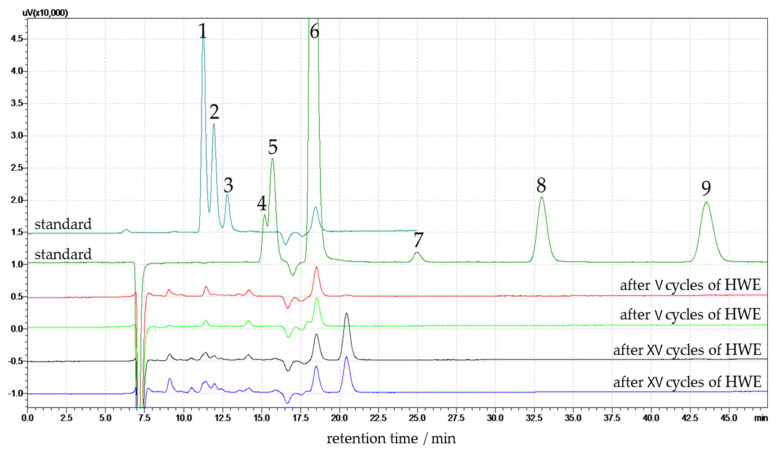
Chromatogram of post-HWE liquid after V and XV cycles performed on hemp and standards. Peaks represent: 1—glucose, 2—xylose, 3—arabinose, 4—formic acid, 5—acetic acid, 6—levulinic acid, 7—ethanol, 8—5-(hydroxymethyl)furfural, 9—furfural.

Hemp biomass was subjected to HWE pre-treatment to solubilize some of the less water-resistant hemicellulosic sugars and other compounds, such as acetic acid, formic acid, levulinic acid, ethanol, furfural, and 5-(hydroxymethyl)furfural (HMF). Therefore, these compounds were also investigated. Monitoring these compounds is essential, as they can act as enzymatic hydrolysis or fermentation inhibitors in downstream biochemical processing. The comprehensive HPLC analysis of the liquid fraction thus provides valuable insight into the HWE treatment of hemp. The HPLC chromatograms comparing standard compounds with post-HWE liquids obtained after V and XV treatment cycles on hemp biomass are presented in Figure 6.

The analysis identifies key monosaccharides and degradation products formed during the HWE process. The standard chromatogram includes all identified compounds with clearly defined peaks for retention time reference. The chromatograms of hemp extracts after V HWE cycles (red and light green lines in the Figure) show very low signal intensities across all analytes. Minor peaks suggest limited hydrolysis of the lignocellulosic matrix at this early stage. Low signals of glucose and xylose, and no degradation products (HMF and furfural) are observed.

Peaks corresponding to glucose and xylose appear between 10 and 13 min, with higher intensity in the XV cycle samples compared to the V cycle samples. Peaks 4, 5, and 6 in the chromatogram correspond to formic acid, acetic acid, and levulinic acid, respectively. They elute at approximately 14.5, 15.5, and 17 min, respectively. These compounds play a crucial role as they are commonly formed as by-products during the thermal degradation of carbohydrates under hydrothermal processes. Their presence in the post-HWE liquid, particularly after XV cycles, indicates increased hemicellulose and partial cellulose breakdown with low polymerization degree and crystallinity index into organic acids. These peaks have a higher area in the samples after XV cycles than in the V cycle samples, reflecting the progressive formation of these degradation products with repeated extraction cycles.

Peak 7, attributed to ethanol, is observed at 25 min in the standard, but no signal is observed in the V and XV cycle samples. Peaks corresponding to 5-(hydroxymethyl)furfural and furfural are detected at approximately 33 and 43 min, respectively. These peaks are not present in any post-HWE samples. The V cycle samples show lower overall peak intensities across all detected compounds than the XV cycle samples. The standard chromatogram includes all identified compounds with clearly defined peaks for retention time reference. The glucose and xylose content in the liquid phase was quantified using an HPLC calibration curve, and the results are expressed as a percentage relative to the dry mass of the native (untreated) hemp. The results are presented in Table 6.

The table presents the percentage content of the two most abundant biomass monosaccharides, i.e., glucose and xylose, determined in the post-HWE liquid phase after V and XV cycles performed on hemp biomass. The mean percentage content and the corresponding standard deviation are provided for each sugar. After V cycles of hydrothermal extraction, the glucose content was 2.3%, and the xylose content was 0.7%. The glucose content increased to 6.8% after XV cycles of HWE. Similarly, the xylose content increased from 0.7% to 2.3% after extending the HWE cycles. The results show an apparent increase in sugar content with the number of HWE cycles.

**Figure 7:**
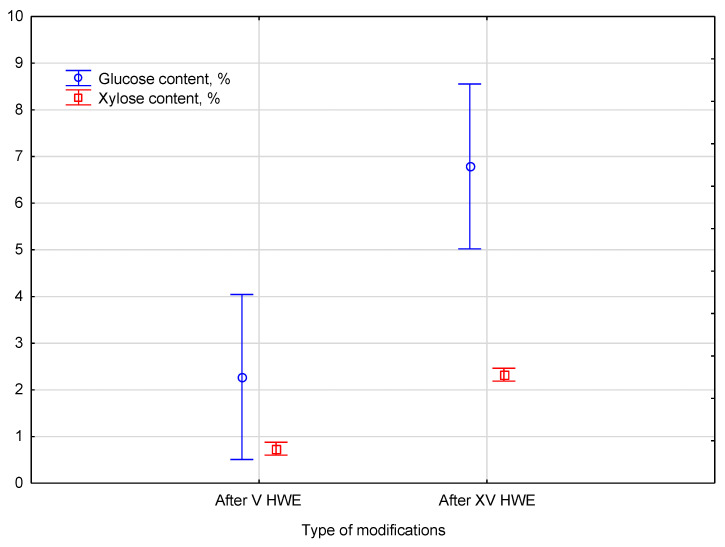
The comparison of glucose and xylose content of the liquid after HWE treatment. Error bars represent the standard deviation (*SD*) of three replicate measurements.

Statistical analysis showed that HWE treatment resulted in variable glucose and xylose content in the liquid phase. It was noticed that glucose and xylose samples each were classified into two homogeneous groups, indicating that the HWE process significantly affected dissolution. Despite varying treatment durations, the sample content remained sensitive to HWE cycles—the comparison of glucose and xylose content of the liquid after HWE treatment is presented in Figure 7.

The liquid phase glucose and xylose content were significantly influenced by HWE treatment. After V extraction cycles, glucose concentration in the liquid phase reached 2.3 ± 0.1%, while xylose concentration reached 0.7 ± 0.1%. After XV cycles, these values increased to 6.8 ± 0.8% and 2.3 ± 0.1%, respectively, showing progressive sugar release. This effect was confirmed by the statistical value of *F*(2, 1) = 1092. The *p*-value of 0.02139 confirms that the differences between the groups were highly significant. After classifying the samples into two homogeneous groups, a post hoc analysis highlighted a clear impact on glucose and xylose content, confirming that the observed increases were statistically robust.

#### 3.3.2. Lignin in Post-HWE Liquid

In addition to HPLC analysis, the liquid fraction obtained after V and XV cycles of HWE was also analyzed using a UV–VIS spectrophotometric method to determine the concentration of soluble lignin. Solubilized lignin, primarily low-molecular-weight aromatic compounds, is released during HWE. Investigating the soluble lignin provides information about the lignin removal during the pre-treatment. It helps assess the potential of the extract for further valorization or its impact on downstream processing. It also helps to provide a deeper understanding of the phenomena occurring during the process. The results are summarized in Table 7.

After V cycles of HWE, the soluble lignin content in the liquid phase was 1.6% of the native dry biomass. Increasing the treatment to XV cycles resulted in a slightly lower lignin content of 1.4%. These results suggest that most easily extractable soluble lignin is released within the first few cycles, with similar results observed upon extended treatment. Additional cycles do not significantly increase lignin solubilization.

**Figure 8:**
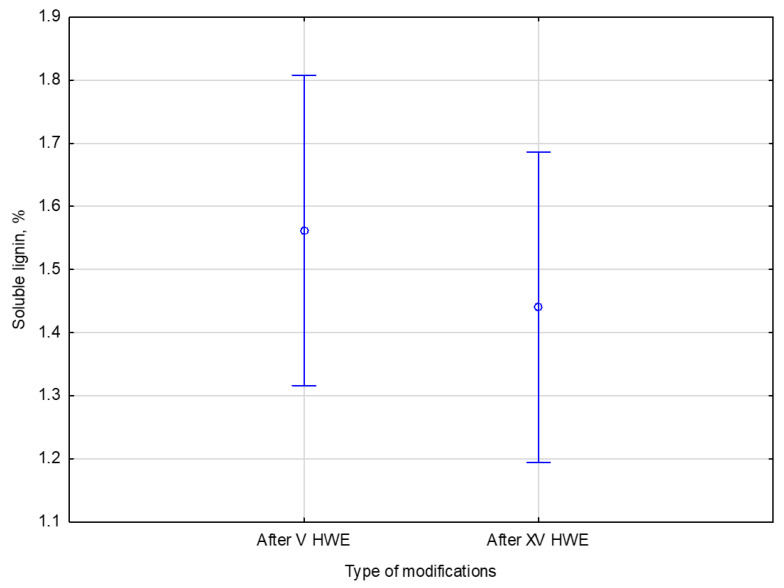
The comparison of the number of cycles and the soluble lignin content. Error bars represent the standard deviation (*SD*) of three replicate measurements.

The statistical analysis sought to determine if extending HWE treatment from V to XV cycles significantly affected the liquid lignin content. No significant differences were found between the two conditions. There was a F statistic of 0.94140 with degrees of freedom 1 and 4, and a *p*-value of 0.38686, well above 0.05, which is generally considered significant. It can be concluded that additional treatment cycles do not increase lignin solubility statistically significantly. The comparison of the number of cycles and the soluble lignin content is presented in Figure 8.

The lignin leaching process appears to reach a state of saturation or equilibrium early. Following V HWE cycles, the soluble lignin content in the liquid phase was 1.6 ± 0.2% of the dry biomass. Extending the treatment to XV cycles resulted in a slightly lower value of 1.4 ± 0.1%. This is supported by the statistical analysis, which showed no significant differences between the two variants. The results suggest that most readily extractable lignin is removed during the initial HWE stage, with subsequent treatment cycles failing to increase its solubility significantly. The equilibrium trend in the Figure illustrates that additional extraction does not enhance lignin removal efficiency.

## 4. Discussion

As part of this study, we investigated how the HWE method affects hemp stalk chemical composition and properties. Hydrothermal treatment, also known as HWE, is a water-based pretreatment that avoids using additional chemical reagents, making it milder compared to acid or alkaline pretreatments. The reaction does not require additional chemicals, but only water at elevated temperature and pressure. The process can be further accelerated by releasing acetic acid from hemicellulose, resulting in a lower pH and intensified autohydrolysis. To assess whether HWE could be used to modify hemp biomass effectively, changes in the content of key components, such as cellulose, lignin, and extractives, had to be analyzed. Optimizing processes to convert biomass into value-added products like biofuels requires understanding these transformations [37,38].

The HWE treatment altered the chemical composition of hemp biomass, consistent with studies conducted on other lignocellulosic plants. Previous studies have shown that the hemicellulose content in biomass decreases with increasing time (number of cycles) of HWE treatment. The lignin content also varies over time: it initially decreases and then increases in later cycles. In contrast, cellulose content follows the opposite trend—rising during the initial stages of HWE and decreasing with prolonged treatment. Extractive content increases during the first few HWE cycles, reaching a peak before declining. These findings were based on studies conducted on Scots pine (*Pinus sylvestris*) [39,40,41]. During the extended extraction cycles, extractive substances and ash contents sharply decreased, which is consistent with the results of our research. The removal of soluble mineral salts is a common outcome of HWE, leading to a reduction in ash content. Similar results were observed in our study. Further biomass processing can benefit from removing these fractions, as extractive substances inhibit enzymes in biochemical processes, and high ash content causes slagging and corrosion in thermochemical processes [42]. Following several cycles, the content of these components stabilizes, suggesting that most of the hot water-soluble components have been removed, which is well documented in science. The stabilization is explained by removing the most soluble, low-molecular-weight compounds in the early cycles. At the same time, the remainder is incorporated deeper into the fiber matrix or has limited solubility. Mass transfer becomes increasingly restricted and structural changes to fibers, such as partial pore closures, further reduce solvent accessibility. Since the overall treatment time is prolonged, additional cycles do not significantly increase extractive removal.

The analysis showed that the relative cellulose content in the solid residue after the HWE process increased proportionally to the number of cycles performed. The increase was probably the result of concentration, resulting from the removal of more thermally labile hemicelluloses and extractive substances. Analysis of the glucose content in the solid phase confirmed that the main cellulose skeleton remained largely intact, which is crucial in bioethanol production. Under moderate thermal conditions, such as HWE, at temperatures ranging from 100 to 180 °C, the degradation of crystalline cellulose is minimal, and the process mainly leads to an increase in its porosity and accessibility. At the same time, the gradual release of glucose into the liquid phase indicates the hydrolysis of amorphous regions of cellulose and other polysaccharides into shorter chains. Maintaining the integrity of cellulose while increasing its accessibility by removing the surrounding hemicellulose-lignin matrix is the primary goal of pretreatment and a prerequisite for efficient enzymatic hydrolysis to glucose.

The hemicelluloses proved to be much more susceptible to degradation during HWE. Analysis of the glucose and xylose content, the primary sugar building blocks of hemicelluloses in hemp, showed their progressive removal from the solid phase and accumulation in the liquid phase. This is consistent with general knowledge about hydrothermal treatment, during which hemicellulose’s amorphous and branched structure makes it much easier to hydrolyze than highly crystalline cellulose [43]. Other researchers obtained similar results. The HWE treatment showed a decrease in hemicellulose with a simultaneous increase in lignin content in wood and bark of different wood species—Douglas fir, Norway spruce, hybrid aspen clones, aspen, willow, eucalyptus—and other biomass—wheat straw, giant miscanthus, agricultural residues, corn stover, hemp stalk and soybean straw [21,44,45,46,47,48]. The decomposition of hemicellulose leads to the formation of not only simple sugars in the form of oligomers and monomers, but also organic acids such as acetic and formic acid. Notably, under the mild conditions used (100 °C), no significant amounts of furfural, a product of further degradation of pentoses and a potent inhibitor of fermenting microorganisms, were detected. Avoiding the formation of inhibitors such as furfural and 5-hydroxymethylfurfural (HMF, from hexoses) is one of the main challenges in designing biomass pretreatment processes, and their formation is strongly dependent on temperature and process duration [26]. The primary goal of HWE is to efficiently extract compounds, such as sugars and extractives, from lignocellulosic biomass without significantly generating inhibitors of downstream processes or changing the cellulose content [49].

The total lignin content in the solid phase remained relatively constant, suggesting that under the conditions used in HWE, no massive delignification occurs. As a complex phenolic polymer, Lignin is generally resistant to hydrolysis under mild hydrothermal conditions such as HWE. Despite this, subtle changes in its structure were observed. Literature studies report that under thermal conditions like HWE, some ether bonds in lignin break down, leading to its partial depolymerization, migration, and redistribution on the surface of cellulose fibers [50]. According to reports, thermal modification, and therefore also using the HWE method, affects the structure of lignin and removes part of the hemicellulose, which can significantly improve the availability of enzymes to cellulose [51]. The differences between AIL and ASL were noted. Our results support the contention that total lignin stability is due to selective solubilization of labile fragments and relative enrichment of resistant pieces. The apparent stability of total lignin content should not be interpreted as a reliable indicator of its resistance to chemical breakdown, but rather as the result of a combination of structural and methodological factors.

According to the research findings, multi-stage HWE is a promising method of modifying hemp biomass due to its reliance only on water as the extraction medium, without adding additional chemicals. This process effectively removes unwanted extractives and ash while increasing the relative cellulose content through selective solubilization and hydrolysis of hemicellulose. The resulting solid residue with increased cellulose availability is an excellent feedstock for bioethanol production. The liquid extract, rich in hemicellulose sugars, can be further processed into other chemicals around the biofuel concept. Further research should focus on optimizing HWE parameters and integrating this process with subsequent conversion stages to maximize the value of the products obtained.

This study offers new insights into the effects of multi-stage HWE on hemp biomass, but there is one significant limitation. We used a single hemp variety based on specific regional conditions in Poland. As such, generalizations should be made with caution. Further studies are necessary to verify whether the observed trends persist and are consistent across hemp varieties and growing conditions. Such comparative studies will be required to confirm that HWE pretreatment can be applied across various industrial applications.

## 5. Conclusions

The research confirms the potential of industrial hemp as a raw material for bioethanol production. HWE pretreatment effectively modified parts of the chemical composition of the hemp stalks, demonstrating a progressive removal of hemicellulose and extractives; this removal of extractives and hemicellulose led to an apparent higher relative proportion of cellulose in the solid residue. Despite this, a statistical analysis confirmed that the absolute cellulose content per initial biomass remained unchanged, indicating that the main cellulose fraction was preserved during HWE.

It was noticed that the overall lignin content showed no statistically significant differences. The HWE process induced a little shift in its composition, with a decrease in acid-soluble lignin (ASL) and a corresponding increase in acid-insoluble lignin (AIL). These changes highlight the complex effects of HWE pretreatment on the plant’s structure. The studies demonstrated that HWE is a promising method for preparing hemp biomass for subsequent conversion processes, effectively concentrating the fermentable cellulose component, for example, in biofuel production.

The multi-stage HWE pretreatment method is an effective and promising method of modifying hemp biomass for biorefinery applications. According to the study, this process selectively removes hemicelluloses and extractives while preserving cellulose, the key fermentable component. The change in lignin composition further illustrates HWE’s ability to alter biomass structure in a way that is beneficial to subsequent processing. It has been shown that HWE is an efficient and sustainable strategy for transforming lignocellulosic biomass into energy and value-added materials by creating a high-value feedstock. The present results were derived from a single hemp variety, so further research involving various varieties and growth conditions will be necessary to validate their general applicability.

## Figures and Tables

**Table 1:** Extractive content in the native and HWE-treated hemp.

Type of Modification	Extractive Content (*SD*), %
Native	3.2 (0.2) ^a^
After V cycles of HWE	2.2 (0.3) ^b^
After XV cycles of HWE	2.0 (0.1) ^b^

*SD*—standard deviation; ^a,b^—homogeneous groups, different letters indicate significant differences.

**Table 2:** Cellulose content in the native and HWE-treated hemp.

Type of Modification	Cellulose (*SD*), %
Native	42.9 (0.8) ^a^
After V cycles of HWE	44.7 (0.3) ^b^
After XV cycles of HWE	46.2 (0.4) ^c^

*SD*—standard deviation; ^a,b,c^—homogeneous groups, different letters indicate significant differences.

**Table 3:** Lignin content in the native and HWE-treated hemp.

Type of Modification	ASL (*SD*), %	AIL (*SD*), %	Total Lignin (*SD*), %
Native	2.6 (0.4) ^a^	21.9 (0.2) ^c^	23.4 (0.4) ^e^
After V cycles of HWE	1.9 (0.2) ^b^	22.4 (0.6) ^c,d^	23.5 (0.5) ^e^
After XV cycles of HWE	2.0 (0.1) ^b^	22.6 (0.3) ^d^	23.6 (0.3) ^e^

*SD*—standard deviation; ^a,b,c,d,e^—homogeneous groups, different letters indicate significant differences.

**Table 4:** Glucose and xylose content in the native and HWE-treated hemp.

Type of Modification	Glucose Content (*SD*), %	Xylose Content (*SD*), %
Native	47.6 (0.4) ^a^	17.9 (0.4) ^b^
After V cycles of HWE	47.9 (1.1) ^a^	19.4 (0.2) ^c^
After XV cycles of HWE	46.9 (1.2) ^a^	18.8 (0.3) ^d^

*SD*—standard deviation; ^a,b,c,d^—homogeneous groups, different letters indicate significant differences.

**Table 5:** Ash content in the native and HWE-treated hemp.

Type of Modification	Ash Content (*SD*), %
Native	3.9 (0.2) ^a^
After V cycles of HWE	3.1 (0.1) ^b^
After XV cycles of HWE	2.7 (0.3) ^c^

*SD*—standard deviation; ^a,b,c^—homogeneous groups, different letters indicate significant differences.

**Table 6:** Glucose and xylose content in the post-HWE liquid after V and XV cycles performed on hemp.

Type of Modification	Glucose Content (*SD*), %	Xylose Content (*SD*), %
After V cycles of HWE	2.3 (0.1) ^a^	0.7 (0.1) ^c^
After XV cycles of HWE	6.8 (0.8) ^b^	2.3 (0.1) ^d^

*SD*—standard deviation; ^a,b,c,d^—homogeneous groups, different letters indicate significant differences.

**Table 7:** The soluble lignin content in the post-HWE liquid.

Type of Modification	Soluble Lignin (*SD*), %
After V cycles of HWE	1.6 (0.2) ^a^
After XV cycles of HWE	1.4 (0.1) ^a^

*SD*—standard deviation; ^a^—homogeneous groups, different letters indicate significant differences.

## Data Availability

The data presented in this study are available on request from the corresponding author.
